# Anatomy in the Training of the Medical Man

**Published:** 1912-01-27

**Authors:** 


### The Hospital

The Workers' Newspaper of Administrative Medicine and Institutional Life, Administration, National Insurance and Health.

No. 1333, Vol. LI. SATURDAY, JANUARY 27th, 1912. PRICE ONE PENNY.

### ANATOMY IN THE TRAINING OF THE MEDICAL MAN.

There are some critics of the medical curriculum who declare that there is not the same need nowadays for a minute knowledge of anatomy as existed in bygone times. These critics urge that the advent of anæsthetics and the evolution of aseptic surgery have permitted the surgeon to take more time, to make a larger incision, and to see his way about, so that he can get a wider view of the part, and does not need to make use of minute points in order to recognise the structure exposed. Following up this line of thought, they argue that, as the medical student has so much to learn which his predecessors never knew, here is a subject that can be lessened in order that the knowledge necessary to pass a qualifying examination should not be more than the ordinary mind can carry. There is something to be said for this argument; though we do not suppose that any examiners would plough a man who did not know the attachments of the muscles of the back, the communications of the petrosal nerves, or the names of the lobes of the cerebellum. And yet it is a theory which would become very dangerous if carried far.

Anatomy is the main foundation upon which all medical knowledge rests. Without some knowledge of this science and of the principles of chemistry and physics physiology cannot be learnt. Again, anatomy can only be learnt by dissecting the human body, and for the majority the opportunity of doing this occurs only once in their lifetime, and is steadily becoming less. Also, it occurs at a period of life in which the student will take advantage of any suggestion that this or that fact need not be learnt, and will extend it to many other facts. The aim of the demonstrator of anatomy is to teach the student how to find his way about the body. The surgeon should know this human topography just as well as the taxi-cab driver knows his way about London. Therefore, any teaching of anatomy which only has a commercial basis in it is bound to fail. To learn only such facts as are known to have some surgical or medical importance is a suicidal proceeding. Many important points will assuredly be missed, and it is better to learn a few unessential facts that can be forgotten later, than to omit to learn essential ones at the only period in which they can be properly acquired. Medicine and surgery as they advance are continually making new use of anatomical facts which originally only had an academic interest. Two instances will suffice. Ten years ago the student, when learning the vertebræ might have read that the laminæ are markedly imbricated in the thoracic region and less so in the cervical region, while in the lumbar region they do not overlap at all, with a complaint at having to remember such unnecessary details. But at the present time any medical man may find it necessary, either for diagnostic or therapeutic reasons, to perform a lumbar puncture, an operation which depends upon this anatomical fact. During recent years the operation for a radical cure of femoral hernia from above Poupart's ligament has come into vogue. This operation depends in that thickening of the periosteum along the inner end of the linea iliopectinea which is known as Astley-Cooper's ligament, which ten years ago had no surgical importance at all.

There is no room for lessening the amount of anatomy to be learnt. The same importance may not be laid upon the structure of individual muscles, and an intimate knowledge of what parts of them are aponeurotic or tendinous; but in their place the connections of the bundle of His must be known, and the nervous system must be learnt in greater detail. It is not sufficient nowadays to know that the deltoid is supplied by the circumflex nerve: for both medicine and surgery the nerve-root and segment supplying this muscle should also be learnt. The same remarks apply to courses of operative surgery. It is the fashion to decry these because the operations done at them are but seldom seen clinically, and because a gastro-enterostomy on a corpse is a different matter from the same operation performed on the living. And yet these courses are the most valuable training there is for teaching that knowledge of human topography and regional anatomy which we have touched on above. A medical practitioner may never be called upon to ligature the popliteal artery, but if he has practised this operation he will be able to incise and drain a deep-seated abscess in that region. These courses of operative surgery have the further advantage of giving to students practice in that manipulation of instruments which is hard to acquire and which is the chief acquisition of the operating surgeon. We have before us two books on operative surgery which illustrate our points. The third English edition of Professor Kocher's work* shows a considerable increase in size upon the second. He starts with the theory that the surgeon must be prepared to cut down upon any artery or nerve or upon any branch of either, not because he will ever have to do so, but because then only can he credit himself with having a complete mastery of anatomy. In other parts of the body the lines along which a structure is approached are indicated rather than described in detail. The book has also an introductory chapter on general considerations, which may well be read by anyone connected with a hospital, surgeons, nursing staff, or secretary's department. The other book is a new edition † of Sir Frederick Treves' small book on operative surgery, which maintains its reputation as an elementary book for students' use at the practical class.

* *Text-Book of Operative Surgery.* By Dr. Theodor Kocher. Third English Edition. Authorised translation, from the Fifth German Edition, by Harold J. Stiles, M.B., F.R.C.S. Edin., and C. Balfour Paul, M.B., F.R.C.S. Edin. 723 pages. 415 illustrations. (London : Adam and Charles Black, 1911.)

† *The Student's Handbook of Surgical Operations.* By Sir Frederick Treves, Bart., G.C.V.O., C.B., LL.D., F.R.C.S., and Jonathan Hutchinson,, F.R.C.S. Third Edition, revised throughout. 512 pages. 162 illustrations. (London : Cassell and Company, Limited, 1911.)

